# Electrical and mechanical switching of ferroelectric polarization in the 70 nm BiFeO_3_ film

**DOI:** 10.1038/srep19092

**Published:** 2016-01-11

**Authors:** Liufang Chen, Zhihao Cheng, Wenting Xu, Xiangjian Meng, Guoliang Yuan, Junming Liu, Zhiguo Liu

**Affiliations:** 1 School of Materials Science and Engineering, Nanjing University of Science and Technology, Nanjing 210094, P. R. China; 2 National Laboratory of Solid State Microstructures, Nanjing University, Nanjing 210093, P. R. China; 3 National Laboratory for Infrared Physics, Shanghai Institute of Technical Physics, Chinese Academy of Sciences, Yu Tian Road 500, Shanghai 200083, P. R. China

## Abstract

Ferroelectric polarization switching and its domain evolution play a key role on the macroscopic electric properties of ferroelectric or piezoelectric devices. Mechanical switching has been reported recently in ~5 nm BaTiO_3_ and PbZr_0.2_Ti_0.8_O_3_ epitaxial films; however it is still a challenge for a mechanical force to switch polarization of a slightly thicker film in the same way as an electric field. Here, we report that the polarization of a 70 nm BiFeO_3_ epitaxial film can be completely switched by a mechanical force, and its domain evolution is similar to that observed with electrical switching. With the gradual increase of the field/force, new domains nucleate preferentially at domain boundaries, the μm-size domains commonly decompose to a mass of nm-size domains, and finally they may reorganize to μm-size domains which undergo 180^o^ polarization switching through multi steps. Importantly, the complete mechanical switching of polarization was also established in the (0 0 1) film with a smooth surface. Furthermore, either upward or downward polarization can be read out nondestructively by a constant current. Our study sheds light on prospective applications of ferroelectrics in the absence of an electric field, such as memory devices and other micro-electromechanical systems.

Ferroelectrics are widely used in electronic, sensing and machinery products, including energy harvesting devices^1^, actuators^2^, tuneable microwave devices^3^, and non-volatile memories^4^. The polarization switching and the corresponding domain evolution are very important for optimizing the ferroelectric and piezoelectric properties of such devices. For example, storing a data bit means increasing the size of one polar region at the expense of another in the non-volatile ferroelectric memory, and thus the motion of domain walls is critical to the applications^5^.

BiFeO_3_ exists in a ferroelectric phase below ~813 ^o^C and a canted anti-ferromagnetic phase below ~370 ^o^C. It is a simple rhombohedrally-distorted cubic perovskite with polarization parallel to one of the four pseudo-cubic body diagonals [1 1 1]^6^. It shows a large remnant polarization (*P*_*r*_ ~100 μC/cm^2^) along the [1 1 1] polar direction. There are four different structural variants in BiFeO_3,_ leading to eight possible polarization directions and three (180^o^, 109^o^ and 71^o^) domain walls^7^,^8^,^9^,^10^. Polarization switching and the corresponding domain evolution of (0 0 1) BiFeO_3_ epitaxial films are important for enhancing their multiferroic properties. Although there are lots of reports about the polarization switching of the BiFeO_3_ film under an electric field, we are still not clear about the domain evolution during the polarization switching period, especially those under a mechanical force.

Strain engineering can modify the properties of thin films using the stress from the substrates or an outer mechanical force. Epitaxial strain is widely used to manipulate the crystal lattice and then to optimize the piezoelectric and multiferroic properties in BiFeO_3_ films^8^,^9^,^10^,^11^,^12^,^13^. In previous studies, a single domain could be induced in the as-grown BiFeO_3_ film by epitaxial strain from the vicinal SrTiO_3_ substrates^10^. Strain may be relaxed through the thickness of a film, however, ferroelectric properties can also be modified thanks to the coupling between strain gradient and polarization known as flexoelectricity. The mechanical switching of ferroelectric polarization has been reported in the 4.8 nm BaTiO_3_ epitaxial film and the 5 nm PbZr_0.2_Ti_0.8_O_3_ film^14^,^15^,^16^. However, it is still a challenge to switch the polarization of the slightly thicker ferroelectric film, especially the BiFeO_3_ film with much larger coercive field than others.

Here the electrical and mechanical switching of ferroelectric polarization are achieved and the corresponding domain evolution is studied in the 70 nm (0 0 1) BiFeO_3_ film with a smooth surface.

## Results

### Polarization orientations and domain structures

**Figure 1:**
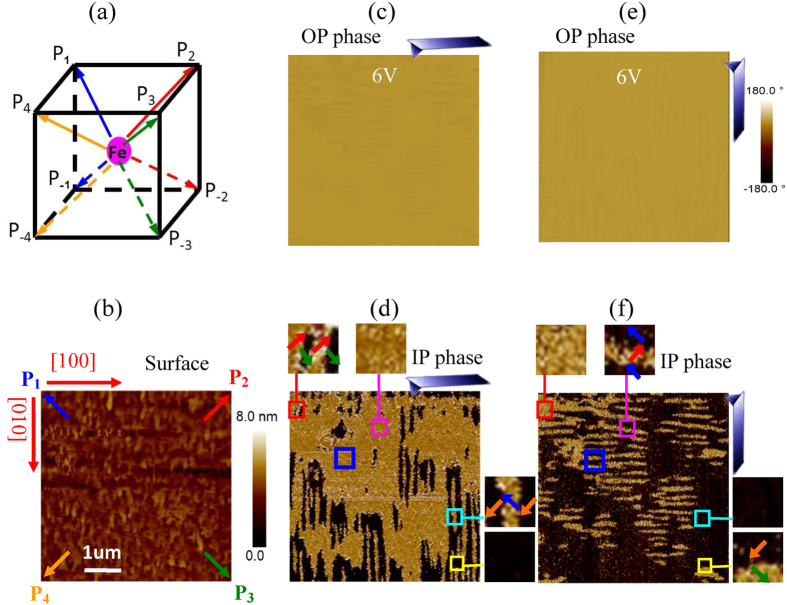
The domain structure of BiFeO_3_ film. (**a**) The sketch of eight possible polarization orientations in a BiFeO_3_ crystal cell; (**b**) the surface morphology, (**c**) out-of-plane (OP) and (**d**) in-plane (IP) phase images of the 6 V polarized regions; (**e**) the OP and (**f** ) IP phase images of the same region observed with a 90^o^-rotated tip, where the [1 0 0] and [0 1 0] are crystal orientations, some domains with P_1_, P_2_, P_3_ and P_4_ are marked in (**d**) and (**f** ), respectively.

The μm-size striped domains with 71^o^ boundaries are introduced in the fully polarized 70 nm BiFeO_3_ film on the (0 0 1) SrTiO_3_ single-crystal substrate with La_0.67_Sr_0.33_MnO_3_ buffer layer. There are eight different polarization orientations along the four diagonal lines of a BiFeO_3_ crystal cell in Fig. 1a^8^,^9^. In the (0 0 1) BiFeO_3_ film, the four upward polarizations (P_1_, P_2_, P_3_ and P_4_) are toward the surface and the four downward polarizations (P_−1_, P_−2_, P_−3_ and P_−4_) are toward the La_0.67_Sr_0.33_MnO_3_ buffered layer. There is a nano-scale smooth surface in the 5 × 5 μm^2^ region of the as-grown BiFeO_3_ film (Fig. 1b). After the as-grown film was polarized by 6 V from the La_0.67_Sr_0.33_MnO_3_ layer (Fig. 1c,e), P_1_, P_2_, P_3_ or P_4_ were introduced (Fig. 1d,f). The tip can probe the polarization to be perpendicular to its cantilever in our system. P_1_ and P_2_ are yellow and P_3_ and P_4_ are black when the cantilever is along [1 0 0] in Fig. 1d, and P_2_ and P_3_ are yellow and P_1_ and P_4_ are black when the cantilever is along [0 1 0] in Fig. 1f^13^. It is found that striped domains with P_2_–P_3_, P_3_–P_4_ or P_4_–P_1_ periodic structure predominate. In previous studies, the polarization orientations and the domain sizes of BiFeO_3_ film were successfully tailored by the static epitaxial strain from the film substrate^8^,^9^,^10^,^11^,^12^,^13^. Although thermal or epitaxial strain did not introduce striped domains in our as-grown film, it stabilized the periodic domain structure after the film was fully polarized.

### Electrical switching of ferroelectric polarization

**Figure 2:**
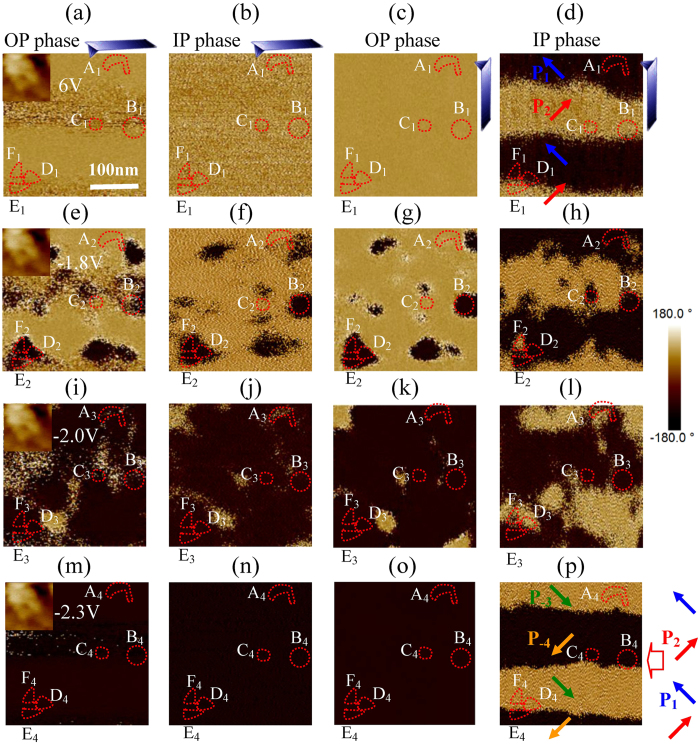
The electrical switching of ferroelectric polarization. The OP and IP phase images of the 6 V polarized area observed by a PFM tip with (**a**,**b**) 0^o^ and (**c**,**d**) 90^o^ rotations, and these phase images of the same area after it was polarized by (**e**–**h**) −1.8 V, (**i**–**l**) −2 V and (**m**–**p**) −2.3 V, where the inset is surface morphology and six independent regions are marked with **A**,**B**,**C**,**D**,**E** and **F**.

New domains nucleate preferentially at the previous domain boundaries under a negative bias. The P_1_–P_2_ striped domains in the blue square of Fig. 1d–f are amplified and shown in Fig. 2a–d, where the inset is the corresponding surface morphology. There is a single domain in A, B, C, D, E and F regions, respectively. After the surface was scanned by a tip with −1.8 V bias, some new domains (e.g. B_2_, C_2_, D_2_, E_2_ and F_2_) appeared at the previous P_1_–P_2_ domain boundaries in Fig. 2e–h^5^. To our surprise, P_1_–P_2_ boundaries were blurry, because the polarization was not unique at domain boundaries within the resolution of PFM. For the −2 V polarized region, the upward polarization becomes downward in over 94% regions (Fig. 2i–l). Many μm-size yellow and black domains do not have clear color contrast with each other, because they decompose to small or even nano-size domains due to various polarization switching routes in neighboring regions (e.g. A_3_)^7^. Nanodomains are beyond the resolution of PFM and thus they show a brown color instead of the yellow or black seen in horizontal PFM images. The brown color did not come from the instrumental phase offset since the μm-size domains outside the −2 V polarized region still show yellow or black colors.

The nano-size domains assembled to μm-size P_−3_/P_−4_ domains in the region of most previous striped domains when the poling field is larger than the coercive field (*E*_C_). After the film was polarized by −2.3 V again, P_−3_ and P_−4_ domains commonly exist in the regions of previous P_1_ and P_2_ domains (Fig. 2m–p), partially because the strain of P_1_–P_2_ striped domains is the same as that of the P_−3_–P_−4_ striped domains. That is to say, the P_1_→P_−3_ or P_2_→P_−4_ 180^o^ switching was realized through two steps (e.g. A_2_(P_1_)→A_3_(P_−1_)→A_4_(P_−3_)) here.

### Mechanical switching of ferroelectric polarization

**Figure 3:**
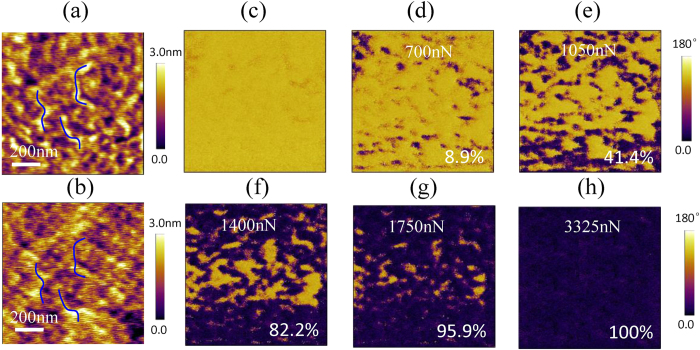
The mechanical switching of ferroelectric polarization. The surface morphology (**a**) before and (**b**) after it was scanned by the PFM tip with 3325 nN mechanical force. The OP phases image of the same region after it was scanned by the PFM tip with a force of (**c)** 140 nN, (**d**) 700 nN, (**e**) 1050 nN, (**f** ) 1400 nN, (**g**) 1750 nN and (**h**) 3325 nN, where the percent is the ratio of area with downward polarization.

A mechanical force applied by the PFM tip can switch ferroelectric polarization through the flexoelectric effect^14^,^15^,^16^,^17^. The surface morphology of our BiFeO_3_ film is nm-scale smooth, as shown in Fig. 3a, which is important for the PFM tip to generate a large stress on the BiFeO_3_ film without inducing irreversible plastic damage. The mechanical switching has been investigated by scanning the 1 × 1 μm^2^ area of the upward polarized domain with the 30-nm-diameter tip under an incremental mechanical force from 700 to 3325 nN, with a corresponding change in the applied stress from 0.25 to 1.18 GPa. Note that, although the maximum local stress is very large, it is still well below the threshold for irreversible plastic damage of the BiFeO_3_ surface (Fig. 3b). In the tip-stressed BiFeO_3_ film, a strain gradient develops mainly along the film’s normal, which introduces an asymmetric distribution of lattice deformation underneath the tip. This gives rise to a local electric field, i.e., the flexoelectric field, pointing to the substrate due to the flexoelectric effect^18^,^19^,^20^. The flexoelectric field is given by *E*_f_ =(f/ε)·(∂e/∂z), where *f* is the flexoelectric tensor, ε is the dielectric constant of the BiFeO_3_ thin film, e is the strain due to the mechanical force of PFM tip, and z is the spatial coordinate along the film’s normal^14^,^15^,^16^,^17^. The *E*_f_ increases with the increase of mechanical force, which breaks the symmetry in the ferroelectric potential of BiFeO_3_ and makes the upward polarizations energetically unfavorable^18^, ^19^, ^20^.

The mechanical switching of polarization and its domain evolution are similar to those of the electrical switching. There is just upward polarization in the original image (Fig. 3c). When the mechanical force is 700 nN, the downward polarized domain starts to nucleate and then propagates. After the force of 700 nN, 1050 nN, 1400 nN, 1750 nN and 3325 nN had been applied by the PFM tip, the downward polarized domains were induced in the 8.9% (Fig. 3d), 41.4% (Fig. 3e), 82.2% (Fig. 3f), 95.9% (Fig. 3g) and 100% (Fig. 3h) region of total area, respectively. The downward polarized domains were stable after the sample had been kept for two days in air, which suggests the complete switching of polarization in 70 nm film.

## Discussion

**Figure 4:**
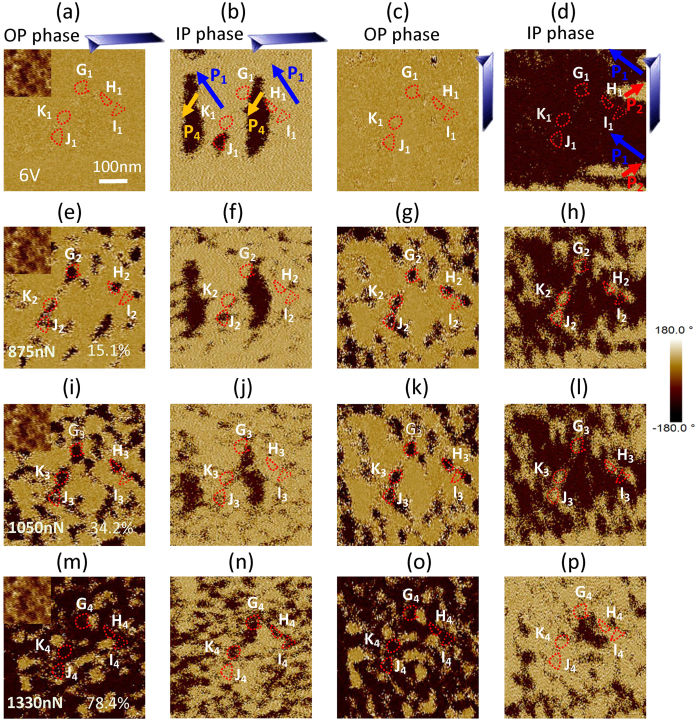
The mechanical switching of ferroelectric polarization. The OP and IP phase images of the 6 V polarized area observed by a PFM tip with (**a**,**b**) 0^o^ and (**c**,**d**) 90^o^ rotations, and the images of the same area after it was polarized by a mechanical force of (**e**–**h**) 875 nN, (**i**–**l**) 1050 nN, (**m**–**p**) 1330 nN, where the inset is the surface morphology, the five independent regions are marked with **G**,**H**,**I**,**J** and **K**, and the percent is the ratio of area with downward polarization.

Table 1 summarizes the possible routes of polarization switching triggered by an electric field (Fig. 2) or a mechanical force (Fig. 4). According to previous phase-field calculation, direct 180^o^ polarization switching is not kinetically favorable because of the high activation barrier. Instead, the ferroelastic relaxation-mediated 180^o^ switching path through 71^o^ switching is expected^21^. Not only the polarization direction but also the direction of lattice distortion changes at 109° and 71° ferroelastic domain walls^7^,^8^,^9^,^10^. There is an easy magnetization plane for the orientation of the magnetic moments, which is always perpendicular to the ferroelectric polarization orientation. Either 109^o^ or 71^o^ polarization switching changes the orientation of the easy magnetization plane^8^,^9^,^10^. The realization of permanent magnetoelectric coupling in the single-phase BiFeO_3_ requires ferroelastic (71^o^, 109^o^) rather than ferroelectric (180^o^) domain switching^21^,^22^,^23^. That is to say, there may exist transient magnetoelectric coupling during the 180^o^ polarization switching which is commonly composed of a 71^o^ and a 109^o^ polarization switching. In the intermediate stage of the 180^o^ switching, there are several possible polarization orientations, and thus tiny domains are the most popular.

Although the ~70 nm BiFeO_3_ film shows a much higher coercive field than that of the BaTiO_3_ film with similar thickness, its polarization was completely switched by a 3325 nN force. Recently, the epitaxial strain from substrate is widely used to tailor domain type or introduce self-polarization in BiFeO_3_^8^,^9^,^10^,^11^,^12^,^13^. Besides, flexoelectricity also plays an important role in the self-polarized direction in BiFeO_3_ film and ceramics^24^,^25^. Nowadays the inhomogeneous strain introduced by PFM tip can switch ferroelectric polarization of the 70 nm BiFeO_3_ film and the mechanical switching of polarization is the same as the electrical switching. In addition to polarization switching, the tetragonal-rhombohedral phase transition can be introduced through an electric field or a mechanical force in a >50 nm BiFeO_3_ film with morphotropic phase boundaries on LaAlO_3_ substrate^11^,^13^,^26^,^27^. Although the mechanical force from AFM tip is large enough to trigger these polarization switching and phase transition indeed, other factors such as the epitaxial strain from substrate or the built-in electric field near the film interface may play an important role on these changes. This work is important for more applications since it is much easier to prepare and measure the ~70 nm ferroelectric films than the ~5 nm films^14^,^15^,^16^,^17^.

The domain evolution due to the mechanical switching is similar to that of the electrical switching. The domain evolution under a mechanical force is clearly observed and its polarization switching routes are identified accordingly. The μm-size P_1_–P_4_ and P_1_–P_2_ striped domains were formed in Fig. 4a–d. There is a single domain in G(P_1_), H(P_1_), I(P_1_), J(P_4_) and K(P_1_) regions, respectively. With the mechanical force increasing gradually from 875 to 1330 nN, new tiny domains nucleate at the boundaries of μm-size domains, and then more and more μm-size domains decompose to nm-size domains due to different polarization switching routes (Fig. 4e–p). Polarization of G, H, J and K first has 71^o^/109^o^ switching and then 109^o^/71^o^ switching^21^,^22^,^23^, e.g. G_1_(P_1_)→ G_2_(P_−2_) → G_4_(P_−3_) and J_1_(P_4_)→ J_2_(P_−3_) → J_3_(P_−2_). As a result, the 180^o^ polarization switching was achieved with two steps. The routes of these regions are shown in Table 1.

**Figure 5:**
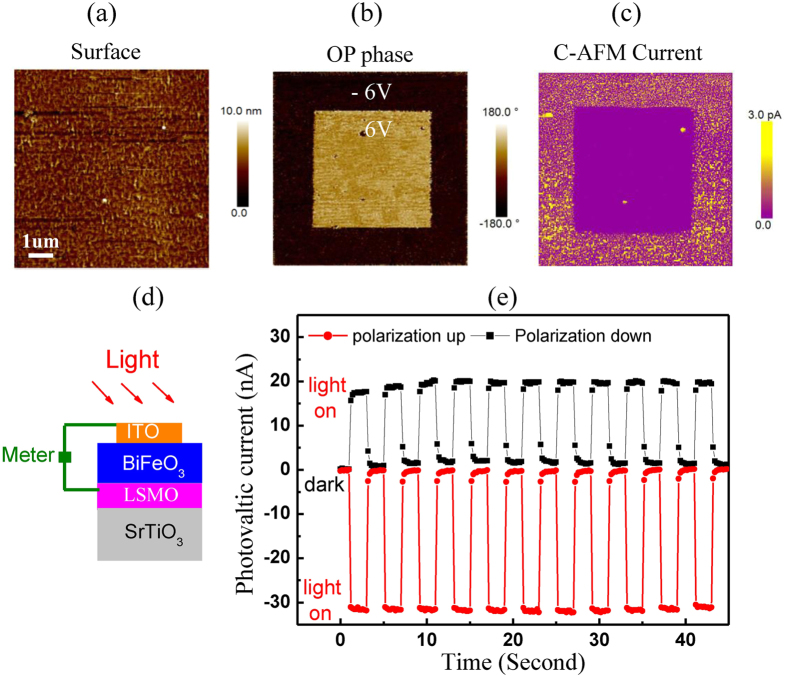
The ferroelectric polarization is nondestructively read out. (**a**) The surface morphology, (**b**) the OP phase image and (**c**) the conductive image with 1 V bias after the film were polarized by 6 V and −6 V bias, respectively. (**d**) The sketch of photovoltaic measurement. (**e**) The photovoltaic current of BiFeO_3_ with upward or downward polarization when light illuminates the BiFeO_3_ surface or not.

Ferroelectric polarization can be nondestructively read-out without another polarization switching in our BiFeO_3_ film. The regions with upward and downward polarization were polarized by a ±6 V tip bias (Fig. 5a–b), and these regions were characterized by a conductive AFM tip with 1 V bias. It is noted that 1 V is much lower than the coercive voltage (*V*_C_) of ±2.3 V which corresponds to the *E*_C_ of 38 kV/mm. The region with downward polarization shows much higher current than the region with upward polarization (Fig. 5c). Therefore the leakage current under a <*V*_C_ voltage can read out information in a nondestructive way^28^,^29^. Oxygen vacancies are typical n-type dopant in BiFeO_3_ film. There is a depletion layer at the interface between the p-type La_0.67_Sr_0.33_MnO_3_ interlayer and the n-type BiFeO_3_ film. The downward polarization increases the leakage current through narrowing the depletion layer at the La_0.67_Sr_0.33_MnO_3_/BiFeO_3_ interface while the upward polarization decreases the leakage current through widening the depletion layer^28^,^29^,^30^,^31^. Furthermore, we measured the intrinsic photovoltaic effect of the BiFeO_3_ films with 300-μm-diameter transparent ITO electrodes which was illuminated by a visible light (~100 mW/cm^2^) (Fig. 5d). The short-circuit current (*I*_sc_) direction is always opposite to the polarization direction in Fig. 5e. The *I*_sc_ after downward poling was 20 nA, whereas the *I*_sc_ after upward poling was about −30 nA^31^. The *I*_sc_ can be switched by polarization flipping. In addition, there is no obvious photocurrent degradation when the *I*_sc_ was measured during eleven on-and-off cycles of the illumination light. Such good retention and high stability over multiple cycles suggest that the polarization status can be nondestructively read out through the analysis of *I*_sc_.

In summary, the electrical switching of polarization in the ~70 nm epitaxial BiFeO_3_ film is similar to that of mechanical switching. The 180^o^ polarization switching is commonly completed with two steps. At first, a μm-size domain decomposes to nano-size domains due to the 71^o^/109^o^/180^o^ polarization switching, and then they merge to a μm-size domain due to the other 109^o^/71^o^/0^o^ switching. Most importantly, the upward polarization can be switched downward with a mechanical force even in the ~70 nm BiFeO_3_ film, and then the polarization status can be nondestructively read out through the analysis of constant current.

## Methods

### Thin film fabrication

BiFeO_3_ (~70 nm) epitaxial films and La_0.67_Sr_0.33_MnO_3_ (~70 nm) buffer layers were grown on commercial (0 0 1) SrTiO_3_ substrates using pulsed laser deposition (PLD) at 630 ^o^C and 16 Pa oxygen pressure inside the chamber. The wavelength, frequency and energy per pulse of KrF excimer laser were 248 nm, 1 Hz and 60 mJ. After that, the as-grown samples were kept at 630 ^o^C and 1000 Pa oxygen pressure for 30 minutes in order to reduce oxygen vacancies, and then they were cooled down at 5 ^o^C per minute to 300 ^o^C. For photovoltaic measurement, indium tin oxide (ITO, In_2_O_3_:SnO_2_=9:1) top electrodes with 300 μm diameter were grown on the BiFeO_3_ film surface at room temperature using PLD.

### Electrical measurements

The surface morphology, out-of-plane (OP) phase, in-plane (IP) phase and amplitude images were studied by using the commercial atomic force microscope (AFM, Bruker multimode 8 or Asylum Research Cypher) with a piezoelectric force microscopy (PFM) mode. The conductive AFM tip is either Bruker MESP-RC with 35 nm tip radius or Nanoworld EFM with 30 nm tip radius. The spatially resolved conductance image was measured with the conductive AFM at 1 V sample bias (Bruker multimode 8). The photovoltaic current is measured with a Keithley 2635 multimeter when the film surface was illuminated by the light of a xenon lamp at 100 mW/cm^2^.

## Additional Information

**How to cite this article**: Chen, L. *et al.* Electrical and mechanical switching of ferroelectric polarization in the 70 nm BiFeO_3_ film. *Sci. Rep.* **6**, 19092; doi: 10.1038/srep19092 (2016).

## Figures and Tables

**Table 1:** Different polarization switching routes of BiFeO_3_ film under an electric field or a mechanical force.

Electrical switching of polarization						
A_1_(P_1_) (6 V)	=	A_2_(P_1_)	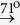	A_3_(P_−1_)	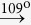	A_4_(P_−3_)(−2.3 V)
B_1_(P_2_)(6V)	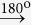	B_2_(P_−4_)	≈	B_3_(P_−4_)	=	B_4_(P_−4_)(−2.3 V)
C_1_(P_2_) (6 V)	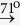	C_2_(P_1_)	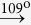	C_3_(P_−4_)	=	C_4_(P_−4_)(−2.3 V)
D_1_(P_1_)(6 V)	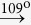	D_2_(P_−4_)	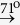	D_3_(P_−3_)	≈	D_4_(P_−3_)(−2.3 V)
E_1_(P_2_) (6 V)	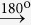	E_2_(P_−4_)	=	E_3_(P_−4_)	=	E_4_(P_−4_)(−2.3 V)
F_1_(P_1_) (6 V)	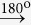	F_2_(P_−3_)	≈	F_3_(P_−3_)	≈	F_4_(P_−3_)(−2.3 V)
Mechanical switching of polarization						
G_1_(P_1_)(6V)	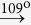	G_2_(P_−2_)	≈	G_3_(P_−2_)	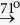	G_4_(P_−3_)(1330 nN)
H_1_(P_1_)(6V)	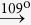	H_2_(P_−2_)	≈	H_3_(P_−2_)	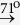	H_4_(P_−3_)(1330 nN)
I_1_(P_1_)(6V)	=	I_2_(P_1_)	≈	I_3_ (P_1_)	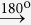	I_4_(P_−3_)(1330 nN)
J_1_ (P_4_)(6V)	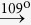	J_2_(P_−3_)	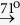	J_3_(P_−2_)	=	J_4_(P_−2_)(1330 nN)
K_1_(P_1_)(6V)	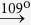	K_2_(P_−2_)	≈	K_3_(P_−2_)	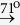	K_4_(P_−3_)(1330 nN)

## References

[b1] Jung J. H. *et al.* Lead-free NaNbO_3_ nanowires for a high output piezoelectric nanogenerator. ACS Nano 5, 10041–10046 (2011). 10.1021/nn2039033 22098313

[b2] Chang C., Tran V. H., Wang J., Fuh Y.-K. & Lin L. Direct-write piezoelectric polymeric nanogenerator with high energy conversion efficiency. Nano Lett. 10, 726–731 (2010). 20099876 10.1021/nl9040719

[b3] Lee C.-H. *et al.* Exploiting dimensionality and defect mitigation to create tunable microwave dielectrics. Nature 502, 532–536 (2013). 24132232 10.1038/nature12582

[b4] Scott J. F. & Paz de Araujo C. A. Ferroelectric memories. Science 246, 1400–1405 (1989). 17755995 10.1126/science.246.4936.1400

[b5] Shin Y. H., Grinberg I., Chen I. W. & Rappe A. M. Nucleation and growth mechanism of ferroelectric domain-wall motion. Nature 449, 881–884 (2007). 17922002 10.1038/nature06165

[b6] Wang J. *et al.* Epitaxial BiFeO_3_ Multiferroic Thin Film Heterostructures. Science 299, 1719–1722 (2003). 12637741 10.1126/science.1080615

[b7] Shafer P. *et al.* Planar electrode piezoelectric force microscopy to study electric polarization switching in BiFeO_3_. Appl. Phys. Lett. 90, 202909 (2007).

[b8] Chu Y.-H. *et al.* Electric-field control of local ferromagnetism using a magnetoelectric multiferroic. Nat Mater 7, 478–482 (2008). 18438412 10.1038/nmat2184

[b9] Zhao T. *et al.* Electrical control of antiferromagnetic domains in multiferroic BiFeO_3_ films at room temperature. Nat Mater 5, 823–829 (2006). 16951676 10.1038/nmat1731

[b10] Chu Y.-H. *et al.* Domain Control in Multiferroic BiFeO_3_ through Substrate Vicinality Adv. Mater. 19, 2662–2666 (2007).

[b11] Zeches R. J. *et al.* A Strain-Driven Morphotropic Phase Boundary in BiFeO_3_. Science 326, 977–980 (2009). 19965507 10.1126/science.1177046

[b12] Cruz M. P. *et al.* Strain Control of Domain-Wall Stability in Epitaxial BiFeO_3_ (110) Films. Phys. Rev. Lett. 99, 217601 (2007). 18233258 10.1103/PhysRevLett.99.217601

[b13] You L. *et al.* Characterization and Manipulation of Mixed Phase Nanodomains in Highly Strained BiFeO_3_ Thin Films. ACS Nano 6, 5388–5394 (2012). 22591450 10.1021/nn3012459

[b14] Lu H. *et al.* Mechanical Writing of Ferroelectric Polarization. Science 336, 59–61 (2012). 22491848 10.1126/science.1218693

[b15] Lu H. *et al.* Mechanically-Induced Resistive Switching in Ferroelectric Tunnel Junctions. Nano Lett. 12, 6289–6292 (2012). 23181389 10.1021/nl303396n

[b16] Guo E. J., Roth R., Das S. & Dorr K. Strain induced low mechanical switching force in ultrathin PbZr_0.2_Ti_0.8_O_3_ films. Appl. Phys. Lett. 105, 012903 (2014).

[b17] Baek S. H. *et al.* Ferroelastic switching for nanoscale non-volatile magnetoelectric devices. Nat Mater 9, 309–314 (2010). 20190772 10.1038/nmat2703

[b18] Baek S. H. *et al.* The Nature of Polarization Fatigue in BiFeO_3_. Adv. Mater. 23, 1621–1625 (2011). 21472789 10.1002/adma.201003612

[b19] Balke N. *et al.* Deterministic control of ferroelastic switching in multiferroic materials. Nature Nanotechnology 4, 868–875 (2009). 10.1038/nnano.2009.293 19893529

[b20] Wen Z. *et al.* Mechanical switching of ferroelectric polarization in ultrathin BaTiO_3_ films: The effects of epitaxial strain. Appl. Phys. Lett. 104, 042907 (2014).

[b21] Zubko P., Catalan G., Buckley A., Welche P. R. L. & Scott J. F. Strain-gradient-induced polarization in SrTiO_3_ single crystals, Phys. Rev. Lett. 99, 167601 (2007). 17995293 10.1103/PhysRevLett.99.167601

[b22] Catalan G., Noheda B., McAneney J., Sinnamon L. J. & Gregg J. M. Strain gradients in epitaxial ferroelectrics, Phys. Rev. B 72, 020102 (2005).

[b23] Catalan G., Sinnamon L. J. & Gregg J. M. The effect of flexoelectricity on the dielectric properties of inhomogeneously strained ferroelectric thin films, J. Phys. Conder. Matter. 16, 2253–2264 (2004).

[b24] Chen X. M. *et al.* Temperature Gradient Introduced Ferroelectric Self-Poling in BiFeO_3_ Ceramics. J. Amer. Cerm. Soc. 96, 3788–3792 (2013).

[b25] Jeon B. C. *et al.* Flexoelectric Effect in the Reversal of Self-Polarization and Associated Changes in the Electronic Functional Properties of BiFeO_3_ Thin Films. Adv. Mater. 25, 5643–5649 (2013). 23897638 10.1002/adma.201301601

[b26] Heo Y., Jang B. K., Kim S. J. & Seidel, J. Nanoscale mechanical softening of morphotropic BiFeO_3_, Adv. Mater. 26, 7568–7572 (2015). 25327302 10.1002/adma.201401958

[b27] Li Y. J. *et al.* Mechnical switching of nanoscale multiferroic phase boundaries, Adv. Funct. Mater. 25, 3405–3413 (2015).

[b28] Jiang A. Q. *et al.* A Resistive Memory in Semiconducting BiFeO_3_ Thin-Film Capacitors. Adv. Mater. 23, 1277–1281 (2011). 21381130 10.1002/adma.201004317

[b29] Yuan G. L. & Wang J. L. Evidences for the depletion region induced by the polarization of ferroelectric semiconductors. Appl. Phys. Lett. 95, 252904 (2009).

[b30] Choi T., Lee S., Choi Y. J., Kiryukhin V. & Cheong S.-W. Switchable Ferroelectric Diode and Photovoltaic Effect in BiFeO_3_. Science 324, 63–66 (2009). 19228998 10.1126/science.1168636

[b31] Yi H. T., Choi T., Choi S. G., Oh Y. S. & Cheong S.-W. Mechanism of the Switchable Photovoltaic Effect in Ferroelectric BiFeO_3_. Adv. Mater. 23, 3403–3407 (2011). 21681986 10.1002/adma.201100805

